# Pheochromocytoma (PC12) Cell Response on Mechanobactericidal Titanium Surfaces

**DOI:** 10.3390/ma11040605

**Published:** 2018-04-14

**Authors:** Jason V. Wandiyanto, Denver Linklater, Pallale G. Tharushi Perera, Anna Orlowska, Vi Khanh Truong, Helmut Thissen, Shahram Ghanaati, Vladimir Baulin, Russell J. Crawford, Saulius Juodkazis, Elena P. Ivanova

**Affiliations:** 1 School of Science, Swinburne University of Technology, Hawthorn, VIC 3122, Australia; jwandiyanto@swin.edu.au (J.V.W.); dlinklater@swin.edu.au (D.L.); pgperera@swin.edu.au (P.G.T.P.); vtruong@swin.edu.au (V.K.T.); saulius.juodkazis@gmail.com (S.J.); 2 Centre for Micro-Photonics, Swinburne University of Technology, Hawthorn, VIC 3122, Australia; 3 Frankfurt Orofacial Regenerative Medicine, University Hospital Frankfurt, Theodor-Stern-Kai 7, D-60590 Frankfurt am Main, Germany; a.b.orlowska@gmail.com (A.O.); s.ghaanati@med.uni-frankfurt.de (S.G.); 4 Departament d’Enginyeria Quimica, Universitat Rovira i Virgili, 26 Avenue dels Paisos Catalans, 43007 Tarragona, Spain; va.baulin@gmail.com; 5 CSIRO Manufacturing, Clayton, VIC 3168, Australia; Helmut.Thissen@csiro.au; 6 School of Science, RMIT University, Melbourne, VIC 3001, Australia; russell.crawford@rmit.edu.au

**Keywords:** mechanobactericidal surfaces, nanostructures, titanium, PC12 cells

## Abstract

Titanium is a biocompatible material that is frequently used for making implantable medical devices. Nanoengineering of the surface is the common method for increasing material biocompatibility, and while the nanostructured materials are well-known to represent attractive substrata for eukaryotic cells, very little information has been documented about the interaction between mammalian cells and bactericidal nanostructured surfaces. In this study, we investigated the effect of bactericidal titanium nanostructures on PC12 cell attachment and differentiation—a cell line which has become a widely used in vitro model to study neuronal differentiation. The effects of the nanostructures on the cells were then compared to effects observed when the cells were placed in contact with non-structured titanium. It was found that bactericidal nanostructured surfaces enhanced the attachment of neuron-like cells. In addition, the PC12 cells were able to differentiate on nanostructured surfaces, while the cells on non-structured surfaces were not able to do so. These promising results demonstrate the potential application of bactericidal nanostructured surfaces in biomedical applications such as cochlear and neuronal implants.

## 1. Introduction

Recent advances in the ability to fabricate large-scale topographical nanofeatures have provided researchers with the opportunity to combat the bacterial contamination of surfaces using a next-generation technology. This technology promises to provide long-lasting and durable mechanobactericidal activity without risking the emergence of bacterial resistance [1,2]. Conventional antibacterial surfaces rely on the diffusive release of antibacterial agents with which the material has been impregnated [3,4]. The leaching of antibiotics and other antimicrobial agents into the environment poses a considerable risk to non-target organisms and may be contributing to an increase in the emergence of multi-drug-resistant pathogenic bacteria. Therefore, antibacterial surfaces that employ non-diffusive techniques are highly preferred and thus have been the focus of a great deal of recent investigation [3].

The ability of certain nanoscale structures to kill bacteria via physico-mechanical means has been investigated over the past few years [5,6,7]. The first observed mechanobactericidal surface was of biological origin; the dense nanopillar array on the surface of the wings of the cicada *Psaltoda claripennis* were observed to selectively kill bacteria [5,6]. Since this time, many new surface structures have been designed, largely modelled upon the surface nanoarchitecture of insect wings, plant leaves, and animal skin [5,8,9]. Such surfaces utilize biomimetic nanoarchitecture in order to achieve regular arrays of nanoscale pillars that are capable of delivering a lethal mechanical force to bacterial cell membranes coming into contact with the surface.

Resistance to bacterial contamination is of particular importance for the manufacture of orthopaedic implants. Nanoengineering of titanium and titanium alloys has been performed in order to generate surfaces possessing nanotopographies that are not only antibacterial, but also display biocompatibility towards human cells [10,11,12]. Considerable progress in implant technologies over the last decade has demonstrated the significance of micro-structured topographies which are able to guide cell growth and tissue development. These surfaces also have the ability to control cell migration and alignment [10,11,12], yet currently the impact of the surface nanoscale topographical features on the growth of mammalian cells is only an emerging area of research, and hence very little is known on this topic. In addition, it is important to proceed with cell-surface interaction investigations in a standardised manner which accounts for superficial modifications of the biomaterials and modification to the culture conditions, all of which may have an impact on the response of cells grown on the studied materials, ensuring greater accuracy of the measurements obtained [13]. Mimicking the surface nanoarchitecture of the dragonfly, hydrothermally etched titanium surfaces have been shown to possess selective bactericidal activity while enhancing the attachment and proliferation of primary human fibroblasts [2]. Another study has shown that the titanium dioxide nanowire arrays inspired by cicada wing surfaces can also be selectively bactericidal, but are capable of guiding human osteoblast-like cell proliferation depending on the presence of a distinct nanostructure [14]. These studies provide examples of the ability of nanostructured titanium surfaces to resist bacterial contamination yet provide a biocompatible scaffold for the attachment and proliferation of mammalian cells.

Pheochromocytoma (PC12) cells are a commonly studied representative of a neuronal cell line which is often used in in vitro studies to examine the degree of differentiation and neurotoxicity commonly associated with neurodegenerative diseases [15,16]. Under common laboratory culture conditions, PC12 cells adhere poorly to culture flasks and prefer to grow while floating in cell aggregates [17]. Therefore, to encourage cellular attachment, tissue culture surfaces are frequently functionalised with a protein. Once adhered to a substratum, PC12 neuron-like cells display growth, proliferation, differentiation, and development of neurite outgrowths [15].

While the presence of micro-scale topographical features on substrata are known to enhance cell attachment [18,19,20], the influence of these bactericidal nanostructured surfaces on the cell behaviour has not been investigated to the same extent, and therefore this study was aimed to fill this gap in existing knowledge. The results demonstrate that mechanobactericidal nanostructures generated on the surface of commercially pure grade titanium can promote the attachment of PC12 cells and enhance the extent of cell differentiation. The cell attachment behaviour on the nanostructured surfaces was compared to that obtained on non-structured titanium surfaces coated with poly-l-lysine.

## 2. Materials and Methods

### 2.1. Preparation of As-Received and Hydrothermally Etched Titanium Discs

Titanium rods, 1 cm in diameter, were cut into 2 mm billets using a Secotom 50 automatic grinder (Struers, Milton, QLD, Australia). Prior to hydrothermal treatment, Ti discs were polished with silicon carbide grinding paper (grit size 1200) and cleaned ultrasonically in MilliQ water, 100% ethanol, 100% acetone, and finally 50% ethanol for 6–8 min each, respectively. This cleaning process was performed to remove organic and inorganic contaminants produced during polishing steps. Afterward, cleaned and polished Ti discs were dried at 37 °C overnight.

Hydrothermal treatment (HTE) of the as-received (AR) titanium billets was performed by immersion in 1 M KOH solution in a Teflon container as described elsewhere [2]. The resulting samples were cleaned with 75% ethanol and sterilised under UV radiation for 30 min prior to experiments being undertaken.

### 2.2. Culturing and Seeding of PC12 Cells

The pheochromocytoma cells (PC12) were purchased from the American Type Culture Collection (ATCC, Manassas, VA, USA)) and were cultured in complete Gibco™ RPMI medium (Thermo Fisher Scientific, Waltham, MA, USA) supplemented with 10% Gibco™ horse serum (HS, Thermo Fisher Scientific, Waltham, MA, USA), 5% Gibco™ foetal bovine serum (FBS, Thermo Fisher Scientific, Waltham, MA, USA), and 1% Gibco™ penicillin/streptomycin (PS, Thermo Fisher Scientific, Waltham, MA, USA) at 37 °C and 5% CO_2_ in a 95% humidified incubator. The medium was changed every two days and passaged accordingly when cell confluence reached 90%.

For each independent experiment, PC12 cells were seeded at a density of 10,000 cells per 100 µL on AR-Ti and HTE-Ti samples. After 1-, 5-, and 7-day incubation periods, the samples were prepared for imaging as described in the following sections. Cell proliferation and total protein count assays were performed after 1 day of incubation to study the attachment patterns. All experiments were approved under the Swinburne Biosafety Project 2014/SBC01.

### 2.3. Cellular Morphology

Scanning electron microscopy (SEM) was used to assess the cell morphology following incubation on AR and HTE Ti surfaces. Prior to SEM analysis, the cells were fixed with 2.5% glutaraldehyde for 25 min. The cells were then dehydrated by passing through a 30%, 50%, 70%, and 100% graded ethanol series for 15 min each. Before imaging, samples were gold sputtered using a NeoCoater MP-19020NCTR (JEOL, Tokyo, Japan). SEM images were taken using a field emission SEM (FESEM) SUPRA 40VP (Carl Zeiss, Jena, Germany) at an accelerating voltage of 3 kV at magnifications of 10,000× for AR and 2000× for HTE samples.

### 2.4. Immunohistochemistry 

The PC12 cells were incubated with the Ti substrate for 1 day. After this time, the samples were initially fixed with 4% paraformaldehyde for 15 min, permeabilised in 0.1% Triton X for 5 min then blocked with 1% Bovine Serum Albumin (BSA) for 60 min. Image-IT^®^ FX Signal Enhancer (Invitrogen, Carlsbad, CA, USA) was also used during the fixation stage to enhance the subsequent fluorescent signals. Samples were then treated with a primary anti-vinculin antibody (Sigma, St. Louis, MO, USA) overnight, followed by goat anti-mouse secondary antibody conjugated with Alexa Fluor 594 (Invitrogen). Actin filaments were visualised by staining the cells with Alexa Fluor 488 conjugated phalloidin (Invitrogen). Cell nuclei were labelled using TO-PRO3 (Invitrogen). To study the extent of cell differentiation after 5 and 7 days of incubation, the anti-nestin antibody (Sigma) was applied as the primary antibody. Samples were then imaged using a Fluoview FV10i microscope (Olympus, Tokyo, Japan) at 60× magnification.

### 2.5. Cell Proliferation

Cell proliferation was determined using the CellTiter 96^®^ Aqueous One Solution Cell Proliferation Assay (Promega, Madison, WI, USA). Tetrazolium was added to the PC12 cell culture at a 10% ratio of the final volume and incubated for 90 min at 37 °C and 5% CO_2_. This allowed for the reduction of MTS (3-(4,5-dimethylthiazol-2-yl)-5-(3-carboxymethoxyphenyl)-2-(4-sulfophenyl)-2*H*-tetrazolium) to formazan, resulting in the formation of a coloured precipitate (purple). The absorbance was recorded at a wavelength of 490 nm using a FLUOstar Omega microplate reader (BMG LABTECH, Thermo Fisher Scientific, Waltham, MA, USA).

### 2.6. Protein Concentration of PC12 

Total protein concentrations were determined using the bicinchoninic acid protein (BCA) assay (Sigma-Aldrich, St. Louis, MO, USA). The PC12 cells were lysed with 150 µL of protein lysis reagent (Sigma-Aldrich, NSW, Australia) and incubated for 15 min at 25 °C. After incubation, the cells were spun down at 1300 rpm for 5 min at 25 °C, then 25 µL aliquots of the supernatant were placed on a 96-well plate (Sarstedt, Germany) together with 200 µL of BCA reagent (bicinchoninic acid solution and copper (II) sulphate pentahydrate 4%). The sample was then incubated for 30 min at 37 °C and the absorbance was recorded at 562 nm using the FLUOstar Omega micro plate reader (BMG LABTECH).

### 2.7. Cell Viability

Cellular viability of PC12 was achieved by labelling them with the LIVE/DEAD Viability/Cytotoxicity Kit (Invitrogen), which is composed of calcein AM and ethidium homodimer-1 for live cell and dead cell staining, respectively. Viable cells were quantified based on the confocal imaging data, expressed as the percentage of non-viable cells over the total population.

### 2.8. X-ray Photoelectron Spectroscopy (XPS)

An X-ray photoelectron spectroscopic (XPS) analysis was performed using a Thermo Scientific K-alpha X-ray photoelectron spectrometer (Thermo Fisher Scientific, Waltham, MA, USA), equipped with a monochromatic X-ray source (Al Kα, hν = 1486.6 eV) operating at 150 W. The spectrometer energy scale was calibrated using the Au 4f7/2 photoelectron peak at a binding energy (BE) of 83.98 eV. During analysis, the samples were flooded with low-energy electrons to counteract any surface charging that may occur. The hydrocarbon component of the C 1s peak (BE = 284.8 eV) was used as a reference for charge correction. Photoelectrons emitted at an angle of 90° to the surface from an area of 700 × 300 μm^2^ were analysed with 160 eV for survey spectra and then with 20 eV for region spectra. Survey spectra were recorded at intervals of 1.0 eV/step, while the region spectra were taken at intervals of 0.1 eV/step. The Shirley algorithm was used to measure the background core level spectra, and chemically distinct species in the high-resolution regions of the spectra were resolved using synthetic Gaussian–Lorentzian components after the background was removed using the Thermo Scientific Avantage Data System software (Thermo Fischer Scientific, Waltham, MA, USA). High-resolution scans were performed across each of the C 1s, O 1s, Ti 2p, K 2p peaks.

### 2.9. X-ray Diffractometry (XRD)

XRD (Bruker D8 Advance) was performed under ambient conditions to determine the degree of crystallinity of the Ti samples. The samples were scanned over a 2*θ* range of 30–85° at a scanning rate of 1 degree per minute using Cu- *K*_α_ radiation (λ = 0.15406 nm).

### 2.10. Scanning Electron Microscopy

High-resolution electron micrographs of Ti discs were recorded using a field emission SEM (FESEM; ZEISS SUPRA 40 VP, Oberkochen, BW, Germany) at 3 kV and 75,000× magnification. To assess the cell morphology of bacteria, titanium discs with adherent bacteria were sputter-coated with gold using a Dynavac CS300 prior to imaging. Characterisation of the surface nanostructure (e.g., edge density, aspect ratio, and tip diameter) was performed using Image J. The colour threshold for binary SEM images was adjusted and the particles were then analysed. The particle analysis allowed an area distribution to be obtained, allowing the determination of the average edge density of the tips per square micron.

### 2.11. Atomic Force Microscopy (AFM)

AFM was used to study the topographical features of the surface at the nanoscale level using an Innova scanning probe microscope (Veeco, Bruker, Billerica, MA, USA) followed by a roughness analysis. The measurements were performed in tapping mode in air to minimise any damage to the tip from the interaction between tip and sample surface. The silicon cantilever used in the tapping mode (Cont20A, Veeco Probes) had a spring constant of 0.9 N m^−1^ and resonance frequency ranging between 18 kHz and 24 KHz. All samples were scanned over a 1 × 1 μm^2^ area to perform a roughness analysis of the surface. To study the surface topography, various surface roughness parameters—average roughness (*S*_a_), root-mean-square roughness (*S*_q_), maximum peak height (*S*_max_), skewness (*S*_sk_), and kurtosis (*S*_ku_)—were calculated using Gwyddion data processing software, and are presented in Table 1 [21]. The results obtained were expressed in terms of their mean values and the corresponding standard deviations following the commonly used protocol [22,23].

### 2.12. Wettability

Surface wettability measurements were conducted using the sessile drop method to measure the static contact angles of MilliQ water on titanium discs. An FTA1000 (First Ten Ångstroms Inc., Portsmouth, VA, USA) instrument was used to measure each water contact angle. An average of at least five measurements was determined for each Ti disc. Each measurement was recorded in 50 images in 2 s using a Prosilica GT camera (Allied Vision, Exton, PA, USA) and the contact angle was then determined using the FTA Windows Mode 4 software. 

### 2.13. Statistical Analysis

Statistical data processing was conducted using the Statistical Package for the Social Sciences, SPSS 21.0 (SPSS, Chicago, IL, USA). Results are presented as the mean ± standard deviation.

## 3. Results and Discussion

### 3.1. Surface Characterisation

**Figure 1:**
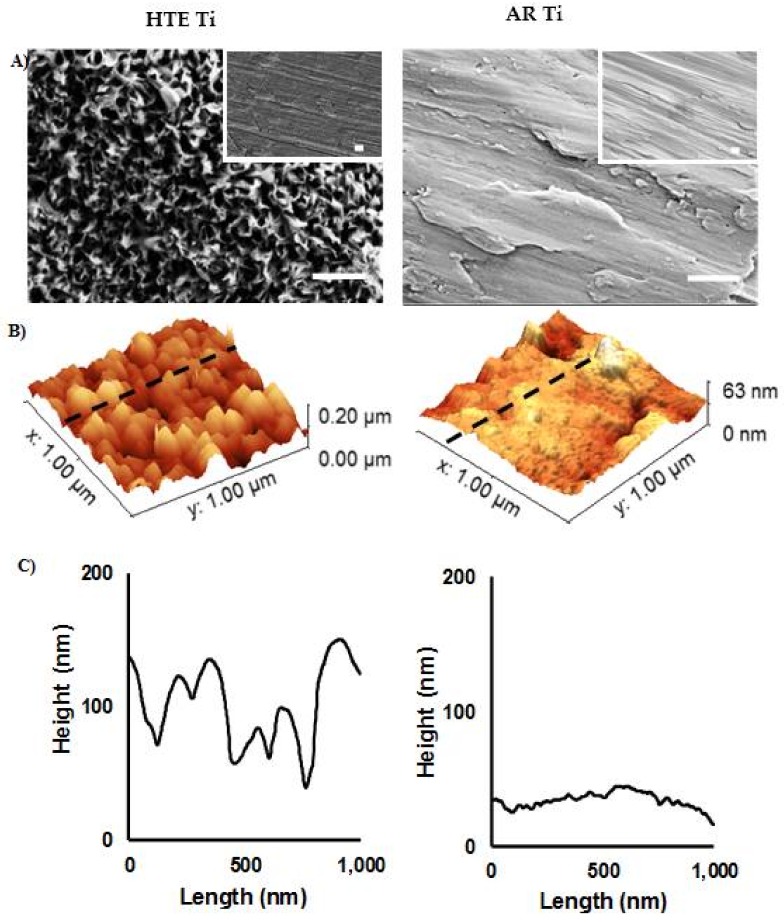
Surface topographic characterisation of as-received and nanostructured titanium. (**A**) SEM images of the surfaces of AR (right) and HTE Ti (left) (scale bar = 400 nm). Insets are images taken at 5000× (scale bar = 2 µm). (**B**) Typical 3D AFM images and (**C**) corresponding surface profiles of AR and HTE Ti surfaces over 1 × 1 µm^2^ scanning areas, showing a significant change in surface nanoarchitecture resulting from the hydrothermal treatment.

The nanostructured Ti surfaces were fabricated using the established technique [2]. Alkaline hydrothermal reactions are commonly used to form nanostructures on titanium because of the reliable nature of the process in fabricating a wide array of titanium dioxide structures, including nanotubes, nanowires, and nanobelts [24]. Hydrothermally etched titanium surfaces were characterised using standard microscopy techniques. SEM micrographs highlight the differences between the AR-Ti and HTE-Ti surfaces on the nanoscale (Figure 1A). HTE-Ti surfaces possessed a network of dense nanoscale features. The change in the surface characteristics resulting from the hydrothermal treatment was quantitatively analysed using AFM. A small (1 × 1 µm^2^) scan area was required to visualise the nanoscale changes to the unmodified Ti as a result of the hydrothermal etching. The comparative surface roughness parameters, including average roughness (*S*_a_), root mean square roughness (*S*_k_), skew (*S*_sk_), and kurtosis (*S*_ku_), are presented in Table 1. These conventional surface roughness parameters indicate that a four-fold increase in surface roughness resulted from the hydrothermal etching process (AR-Ti *S*_a_ = 6.2 ± 2.5 and HTE-Ti *S*_a_ = 26.5 ± 3.8 nm). This finding is confirmed by the 3D reconstruction of the AFM scans and the associated line profiles (Figure 1B, C). The height of the nanoscale structures can be seen to vary considerably.

**Figure 2:**
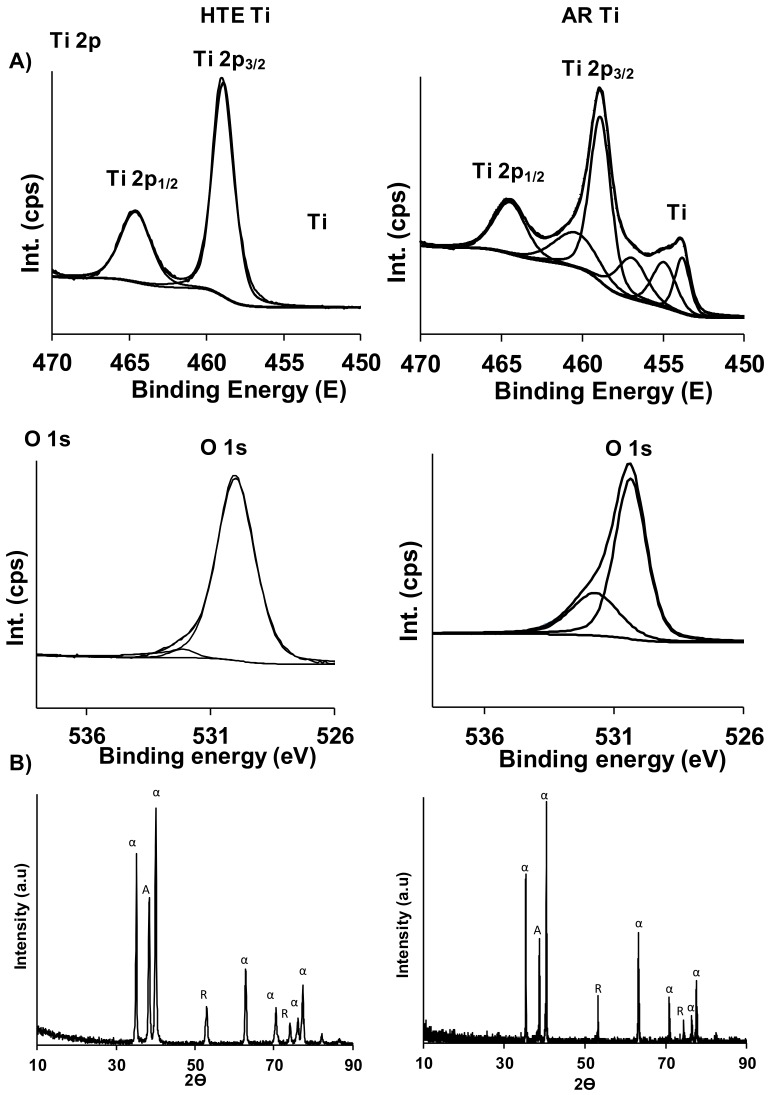
Surface chemistry and crystallinity characteristics of titanium surfaces. (**A**) XPS spectra of Ti 2p, and O 1s for the as-received (right) and HTE (left) titanium substrata. (**B**) X-ray diffractograms demonstrating the crystalline phases present on as-received (right) and HTE (left) substrata.

The surface chemical analysis of the AR and HTE Ti surfaces confirmed the formation of titanium dioxide (TiO_2_) nanofeatures. XPS was used to define the chemical composition of both surfaces. High-resolution scans of the Ti 2p region revealed that the surface nanotopography (post-processing) was predominantly TiO_2_, while the X-ray diffractograms provide confirmation of a surface with enhanced crystallinity, evidenced by an increase in the anatase (A) phase (Figure 2). No significant differences were observed in the surface chemistry of the AR and HTE-Ti samples. The hydrothermal etching of the Ti substrata resulted in an increase in the surface wettability, decreasing the water contact angle from 58.9° to 23.1°. This increased degree of surface wettability is most likely a result of the formation of nanostructures on the Ti surface [25].

The physical and chemical characteristics of a surface are particularly important in ensuring the successful biointegration of an implant material, as these two factors directly influence the initial interactions between human tissue and foreign material being implanted into the body [26]. Surface chemistry, in particular, determines the adsorption of protein from bodily fluids. Of the hydrothermally-modified Ti surfaces used in the present study, cells were primarily exposed to titanium dioxide, and the crystalline structure—as identified by XRD—was mostly anatase (Figure 2). Surface chemical characteristics between the non-structured control and hydrothermally-treated surfaces did not vary significantly enough to comment on the influence of surface chemistry on the proliferation and differentiation of PC12 neuron-like cells.

### 3.2. The Proliferation of PC12 Cells on Titanium Surfaces

The impact of nanostructures on the attachment and proliferation of mammalian cells on surfaces has been well-documented [18,19,20]; however, very little understanding has been obtained regarding the nature of the interaction taking place between neuron-like cells and the modified titanium surfaces possessing bactericidal nanostructures. As previously stated, PC12 cells adhere poorly to the smooth surfaces of culture flasks, preferentially growing as cellular aggregates in suspension [17]. To encourage cellular attachment onto the surface of tissue culture flasks, they are often functionalised with protein, such as collagen. Only once attached to a substrate are the PC12 cells able to proliferate over the surface in a differentiated form [27].

**Figure 3:**
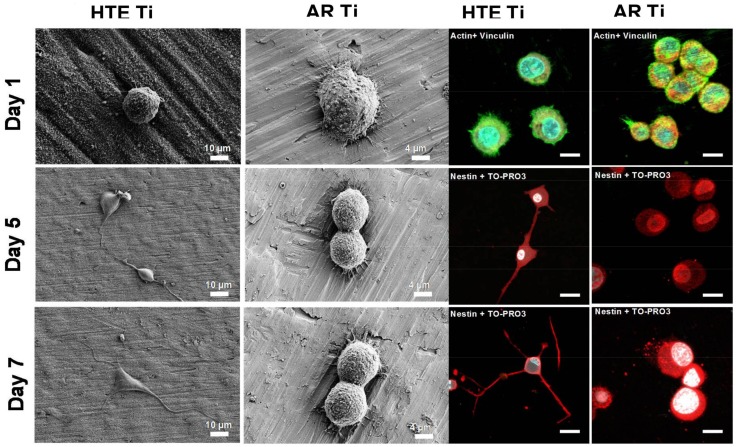
PC 12 cell morphology on HTE and AR Ti surfaces. The PC 12 cells were only able to differentiate on the surfaces of the nanostructured substrata in the presence of nerve growth factors (NGFs). No differentiation was observed on the non-structured AR surfaces. Expression of nestin was observed on the differentiated PC12 cells grown on the HTE-Ti substrates over a 7-day period. Immunohistochemical staining (nestin, red) of the PC12 cells showed that the cells could be differentiated on the surface of the nanostructured substrates. The cells exhibited an enhanced neurite elongation and expression of nestin, as seen on days 5 and 7. The PC12 cells grown on the AR surfaces were not observed to differentiate (scale bar = 3 µm). Actin (green) and vinculin (red) were labelled on day 1 to enable the determination of cell attachment patterns.

To determine the mechanisms by which the PC12 cells underwent proliferation and differentiation on the HTE-Ti surfaces, the PC12 cells were seeded onto both the nanostructured and non-structured substrata and visualised after 1, 5, and 7 days of incubation. The nanostructured Ti surfaces were not coated with protein in order to effectively determine their suitability as substrata for initial anchorage. After day 1, analysis of the SEM and corresponding confocal laser scanning microscopy (CLSM) images showed that the PC12 cells were successfully attached onto the surface (Figure 3). At day 5, the PC12 cells exhibited a change in morphology, with the beginnings of neurite outgrowths being evident, which is an indication of successful differentiation. At day 7, the PC12 cells exhibited a large neurite growth, extending over the nanostructures. In contrast, the PC12 cells seeded onto the non-structured AR surfaces attached and proliferated over the surface, but failed to differentiate and produce neurite outgrowths, even in the presence of nerve growth factors (NGFs), retaining their rounded morphology. The expression of nestin—the type VI intermediate filament protein that is produced in nerve cells [28,29]—can be seen in the CLSM images (stained red) at day 5 and 7 only for the differentiated cells on the HTE-Ti surfaces. It is evident that the nanostructures present on the HTE-Ti substrates provided focal adhesion points for the neuron-like cells to attach and provide further directional cues for growth and differentiation. The results presented here provide strong evidence as to the biocompatibility of the nanostructured Ti surfaces, with the surface nanostructures clearly affording the surface characteristics that are beneficial for the growth and proliferation of nerve cells. Such surfaces are suitable for use in implant applications without the need for additional surface coatings to encourage the attachment and differentiation of neurons.

**Figure 4:**
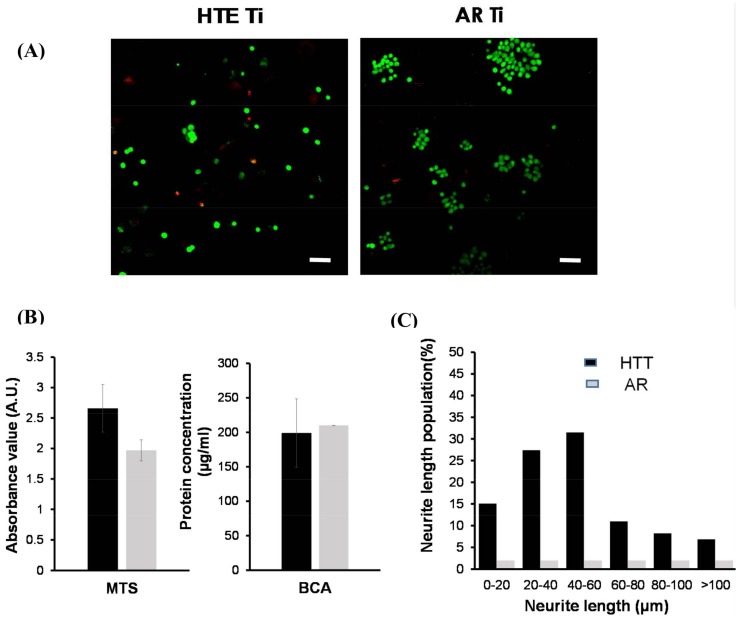
PC12 cell viability and differentiation on HTE and AR Ti surfaces. (**A**) Confocal laser scanning micrographs showing viable (green) and nonviable (red) bacterial cells on the HTE- Ti and AR-Ti surfaces. The majority of PC12 cells still survived on both surfaces after one day of incubation, and the mechanobactericidal surface of HTE-Ti did not have any killing effect towards PC12 cells (scale bar 50 µm). (**B**) Day 1, PC12 cell attachment and proliferation on HTE-Ti and AR surfaces. MTS (3-(4,5-dimethylthiazol-2-yl)-5-(3-carboxymethoxyphenyl)-2-(4-sulfophenyl)-2*H*-tetrazolium) results suggest that the HTE-Ti facilitated cell attachment. No differences were detected in the total protein content (BCA, bicinchoninic acid protein assay) of the two samples tested. (**C**) PC12 cell differentiation on HTE-Ti. PC12 cells present on HTE-Ti surface exhibited enhanced neurite growth for 7 days. The cells grown on the AR surfaces were not able to undergo differentiation.

The viability of PC12 cells attached onto nanostructured substrata was estimated by applying a range of qualitative and quantitative bioassays. Live/dead staining yields two-colour discrimination of the population of live and dead cells. Green-fluorescent calcein-AM indicates the presence of intracellular esterase activity, whereas red-fluorescent ethidium homodimer-1 indicates loss of membrane integrity. Fluorescent micrographs in Figure 4A show the proportion of live (green) and dead (red) cells on the HTE and AR substrata. The HTE-Ti surfaces supported the attachment and growth of neuron-like cells, following one day of incubation. In similarity with the smooth control, the numbers of dead cells were negligible (7.4% and 2.0%, respectively). MTS cell proliferation and viability assays (Figure 4B) give a quantitative measure of metabolically-active cells, and showed that the cells attached to all substrata remained viable for the maximum 1-week incubation period. Absorbance readings of the reduced MTS compound highlighted that the surface modification process provided a substratum that actually encouraged cellular proliferation, with increased levels of formazan being present in the culture wells containing the PC12 cells on HTE-Ti; however, no significant difference was detected in the total protein concentration for cells coming into contact with the nanostructured and non-structured substrata. Finally, in Figure 4C, neurite growth also serves to demonstrate the health of attaching nerve cells, with measurements of neurite outgrowths at 7 days showing that ~30% of the cells exhibited growths of 40–60 µm in length, whereas those on non-structured surfaces did not produce growth extensions at all and failed to differentiate, which is typical for these cells on surfaces not coated with protein.

Previously, it was demonstrated that osteoblast-like cells responded to HTE-modified Ti surfaces through an enhanced cellular attachment and proliferation compared to non-structured titanium substrata [25]. On similar surfaces, primary human fibroblasts were shown to successfully attach and proliferate, with the surface providing sufficient anchorage points and cues for enhanced growth and elongation of filopodia [2]. Furthermore, the behaviour of mesenchymal stem cells (MSCs) on Ti surfaces fabricated by the same method as used in this study was also observed [30]. Significantly, compared with non-structured Ti substrata, MSCs cultured on HTE-Ti substrata displayed significantly higher proliferation and differentiation levels of alkaline phosphatase and osteocalcin after 7- and 14-day cultures, respectively. Often, the biocompatibility of titanium implants is studied using oral-derived cell lines due to the heavy use of titanium to treat dental caries [31,32,33]. Upon its placement in the jaw, a dental implant must encounter osteoblasts from the bone, epithelial cells and underlying fibroblasts. Therefore, in a recent systematic study of human gingival fibroblasts, epithelial cells, and osteoblasts grown on alkali-hydrothermally etched Ti, it was shown that the osteogenic activity of osteoblasts was enhanced, and the adhesion activity of human epithelial cells and fibroblasts was promoted as compared to smooth surfaces [31]. These results, and results obtained in the current study, suggest that the nanostructured titanium substrata have great potential for inducing growth and differentiation in multiple cell lines, including osteoblasts and neurons, and that the approach presented here may be exploited to fabricate titanium-based implants.

Enhanced osseointegration has frequently been observed in cases where modified Ti substratum have been implanted into an animal model, and under conditions where the Ti implants are exposed to normal bodily fluids and proteins [34,35,36]. A study investigating the implantation and response of a modified Ti surface possessing titanium dioxide nanorods in rabbit femurs achieved better osseointegration and higher rates of bone tissue apposition of the nanostructured Ti, compared to classically treated (acid etched, grit blasted) micro-rough Ti surfaces [34]. Other studies have demonstrated the significantly increased primary and secondary stability of nano-modified titania implants and increased new bone formation [37,38]. More interestingly, although it has been confirmed in vitro, in vivo models have also established the influence of the *shape* of the nanostructures in enhancing osseointegration [39,40]. Over a range of specific nanomorphologies investigated in vivo in rats, nanoleaves—being a network of vertically aligned, non-periodic “leaf-like” structures—promoted increased osteoblast cell proliferation, alkaline phosphatase activity, and collagen synthesis with reduced inflammatory responses over other shapes investigated. An inflammatory cytokine analysis of both chronic and acute cytokines revealed no significant increase due to the presence of nanoscale features as compared to smooth controls [40]. The similar Ti nanomorphologies presented in the literature—which have been studied in vivo—implies that the Ti surface modifications achieved in this study will also promote both antibacterial activity and osseointegration as an implant, although here we have tested neuronal response to the surface, which is the first study of its kind according to our knowledge.

The biocompatibility of materials is a prerequisite for the manufacture of orthopaedic implants and for use in medical devices. Although Ti surfaces are chemically inert, surface suitability and functionality can be greatly altered through changes to surface chemistry or topography. Current approaches to the surface modification of implants do not make the distinction between mammalian and bacterial cells, and may easily discourage or encourage the attachment and growth of both. Micro-nanoscale modification of surfaces can also have a differential impact on the formation of focal adhesion points for mammalian cells, even though the surface is “antibiofouling” in nature. With this in mind, the design and fabrication of a bactericidal medical implant material that can support the proliferation and differentiation of neuron cells is particularly significant. Moreover, because the surfaces in this study capitalise on the mechanical interactions between cells and surfaces and because they can be easily fabricated using a simple, scalable process, the translation of this research on hydrothermally treated titanium surfaces into a commercial product should readily be achieved.

## 4. Conclusions

Commercial-grade Ti was successfully modified to create surfaces with an array of bactericidal nanofeatures. The effect of the nanostructures on PC12 cells was studied using cell viability assays and cellular morphology to assess cell–substrata interactions. It was shown that the neuron-like cells preferred to attach to the HTE-Ti substrata compared to the non-structured surfaces, even without a pre-coating of poly-L-lysine. Once attached onto the nanostructured titanium surfaces, PC12 cells demonstrated enhanced proliferation and differentiation. This study therefore confirmed the potential of nanostructured titanium surfaces to induce PC12 cells differentiation into neurons, without the need of surface pre-treatments.

## Figures and Tables

**Table 1:** Surface chemical, topological, and physico-chemical characteristics of as-received and nanostructured titanium surfaces obtained by X-ray photoelectron spectroscopy (XPS), atomic force microscopy (AFM), and water contact angle measurements. AR: as-received; HTE: hydrothermal treatment.

Samples	Chemical composition (%)				Wettability	AFM (1 × 1 µm^2^)			
	C	O	Ti	K	Water Contact Angle (°)	*S*_a_ (nm)	*S*_q_ (nm*)*	*S*_sk_	*S*_ku_
HTE	13.5 ± 0.6	56.7 ± 0.5	22.7 ± 0.2	7.3 ± 0.1	23.1 ± 4.3	26.5 ± 3.8	33.9 ± 5.6	−0.2 ± 0.1	0.2 ± 0.1
AR	25 ± 0.3	48 ± 0.5	27 ± 0.3	27 ± 0.3	58.9 ± 4.8	6.2 ± 2.5	8.8 ± 3.9	0.1 ± 0.9	2.5 ± 1.1
